# A randomized controlled trial for families with preschool children - promoting healthy eating and active playtime by connecting to nature

**DOI:** 10.1186/s12889-016-3111-0

**Published:** 2016-06-13

**Authors:** Tanja Sobko, Michael Tse, Matthew Kaplan

**Affiliations:** Institute of Human Performance, The University of Hong Kong, 303A, 3/F Bldg. for Interdisciplinary Research, 5 Sassoon Rd, Pokfulam, Hong Kong; Department of Agricultural Economics, Sociology, and Education, The Pennsylvania State University, University Park, Pennsylvania USA

**Keywords:** Connectedness to nature, Healthy eating, Active playtime, Toddler

## Abstract

**Background:**

Promotion of healthy lifestyles in children focuses predominantly on proper nutrition and physical activity, elements now widely recognised as essential for a healthy life. Systematic reviews have shown that nature-related activities also enhance general well-being as reflected in increased physical activity, a healthier diet, reduced stress and better sleep. Recent research suggests that many young children in Hong Kong between the ages of two and four in Hong Kong are more sedentary than recommended and seldom participate in active play, placing them at risk of becoming overweight or obese. The proposed project aims to investigate whether connecting families to nature positively influences physical activity (i.e., active playtime) and healthy eating routines in children aged 2 to 4.

**Methods:**

We recently conducted a pilot study in Hong Kong to develop a programme, Play & Grow, based on the most successful evidence-based international preschool interventions. In addition to adopting the healthy eating and physical activity elements of these interventions, this project will additionally include a third novel element of Connectedness to nature: discovering nature through games and awareness of sounds, touch, smells, and temperature. To test the effectiveness of this modified intervention, a randomised controlled trial (RCT) involving 240 families with children aged 2 to 4 will be conducted. Families and children will take part in weekly one-hour activity sessions for 10-weeks. Lifestyle-related habits will be assessed before and immediately after the 10-week intervention, with follow up testing at 6 and 12 months’ post intervention.

**Discussion:**

A novel measuring tool created specifically for assessing Connectedness to nature, Nature Relatedness Scale (NRS), will be validated and tested for reliability prior to the RCT. The results of the RCT are intended to be used to understand which components of the intervention are most effective. The objectives of this project will be achieved over a 30-month period and will contribute to the research that examines key components of successful healthy lifestyle promotion programmes during early childhood. We predict that the inclusion of Connectedness to nature will significantly improve recognised preschool interventions. Finally, the aim of targeting family involvement will hopefully increase the sustainability of longer-term lifestyle modifications in children.

**Trial registration:**

ClinicalTrials.gov, NCT02715544. Registered 22 March 2016

## Background

Proper nutrition and physical activity are essential for a healthy life [1]. Modern-day children practice unhealthy habits and routines; they play indoors, engage in sedentary activities, eat poorly and get insufficient sleep. Lifestyles are generally becoming unhealthier worldwide, and Hong Kong is no exception [2, 3]. The urban environment is criticised for being ‘toxic’, promoting the ‘nature-deficit’ phenomenon, and for stimulating less physical activity in young children [4, 5]. In Hong Kong, the majority of people live in densely populated areas, moving within a concrete labyrinth with little exposure to nature. The prevalence of overweight and obesity amongst Hong Kong children has become an increasingly prominent public health concern. A recent study of the Hong Kong population, conducted in 2013, reported that children as young as 6 months were overweight/obese and by the age of 2 years of age almost 5 % of the territory’s children were overweight/obese [2, 3, 6]. Any routines, healthy and unhealthy that get introduced in early childhood may stay for life, strongly suggesting that interventions promoting healthy living should start as early as possible [7]. For these reasons, we have chosen to target families with children aged 2 to 4 in our proposed research. It is widely accepted amongst the caregivers that preschool children are sufficiently active and as a result of this belief is that preschool children are often overlooked in physical activity research [7]. This belief, however, does not always reflect the real situation. The family setting provides the most powerful influence on preschool children; consequently, many of the risk factors for obesity in the preschool years are rooted in the family context [8]. Parents have the capacity to impact their children’s emerging food choices with their knowledge of nutrition, and their parenting style and modelling [9]. The strongest predictor of vegetable consumption in 2–6 year old children is parental consumption, and when it comes to negative eating behaviour in this age group, this can be modified if handled properly [10]. Furthermore, children with active parents also tend to be more active [11]. In Hong Kong, parents often adopt inappropriate strategies in managing the physical activities and dietary habits of their children, such as encouraging overeating i.e. "force feeding", and allowing children to watch TV while eating, etc. [2, 3, 6]. We therefore intend to conduct a family intervention, targeting children’s caregivers, as it is well-confirmed that they are responsible for introducing the lifestyle routines in young children and thus play one of the biggest roles in influencing their habits [12]. Although grandparents and domestic helpers and are also highly involved in taking care of the children of in Hong Kong, we targeted families with parents as the primary caregivers and not domestic helpers in order to test our hypothesis in a more controlled and homogenous group. The tools which are often utilised by parents to manage some problematic behaviour in children (e.g., food fussiness) [13] are documented to firstly improve *general* parenting and secondly, the parenting practices in *specific* situations [13]. A recent Cochrane review reported only a marginally significant improvement in body weight after some interventions and little to no effect on dietary or physical activity behaviours [14]. Hence, developing new lifestyle intervention programs for preschool children and testing these is highly relevant.

Time spent in nature has also proven to be beneficial for health and wellbeing. The systematic reviews conducted recently have pointed out that nature-related activities enhance general well-being and lead to increased physical activity, a healthier diet, improved sleep and reduced overall stress [15–18]. Worldwide, the most current programmes promoting a healthy lifestyle for children focus almost exclusively on diet and physical activity [19], but few take into consideration a family’s interactions with nature. We suggest that this emerging research area, known as Connectedness to nature or Nature Relatedness [20], is highly related to healthy lifestyle. Unhealthy lifestyle is often explained by environmental factors, while more time spent outdoors in a natural environment is reflected in healthy life patterns [15, 21].

We recently conducted a pilot study in Hong Kong, Play & Grow learning, which is based on the most successful preschool interventions from Sweden and Australia [22, 23]. The results of the pilot work helped formulate the basis of the present project by incorporating the healthy eating and physical activity elements of Play & Grow to which we also included a third novel element; Connectedness to nature. The proposed project will examine this modified Play & Grow intervention aimed at promoting healthy lifestyle in preschool children (aged 2 to 4) through the combined intervention of healthy eating, active play and connectedness to nature habits to children and their families.

The strategies outlined in this study are particularly appropriate to Hong Kong for several reasons. First, most Hong Kong children in primary schools fail to reach the physical activity levels recommended by the health authorities, and moreover, their increased sedentary habits lead to deterioration in health [2, 3, 24]. Second, Hong Kong children’s food habits fail to meet recommended nutritional standards: the major source of energy intake derives from calorie-dense foods, saturated fats and sugar [6], negatively influencing the territory’s obesity levels [6]. Third, this unhealthy lifestyle has negative consequences not only on general well-being but also academic performance; an issue of great concern to Hong Kong parents. It is therefore hoped that the proposed project will bring sustainable health benefits to Hong Kong’s population in a long run. These positive changes and benefits will hopefully in turn lead to reduced prevalence of obesity amongst children in Hong Kong, and a concomitant decrease in healthcare related costs.

In addition, this pioneering project will have scientific value in development of several important protocols and measurement instruments, including our Nature Relatedness Scale. Existing instruments for nature relatedness are applicable only to adults and have not yet been tested in Asia; thus, our new instrument will contribute to international research in this field, which has been relatively sparse to date.

### Conceptual influences

**Fig. 1:**
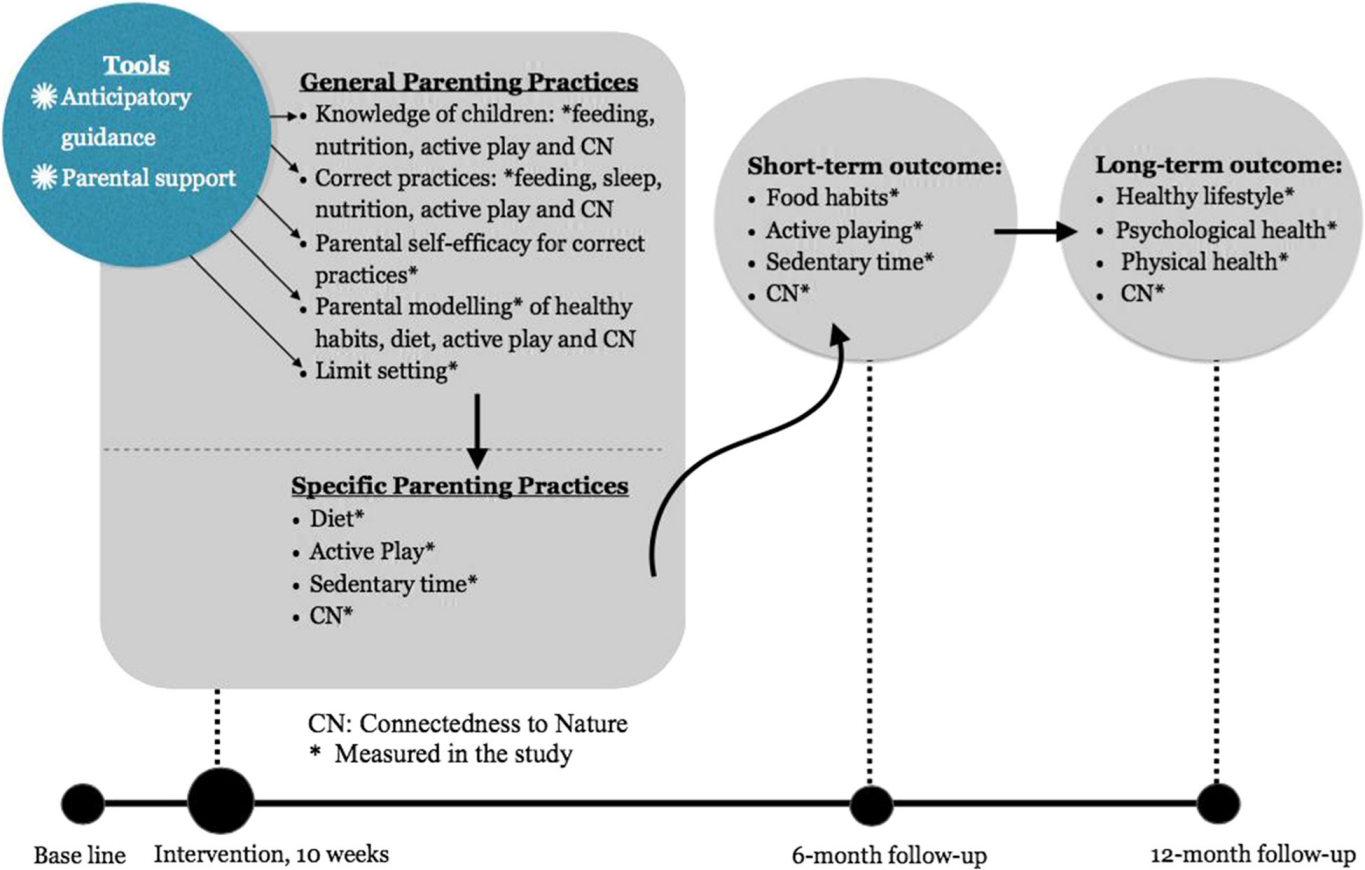
Conceptual modelling of the relationships between the parenting and the outcomes of healthy lifestyle in children

Children’s optimal development is promoted by active family involvement (Patterson’s Social Interaction Learning Theory) [25]. One of the most effective lifestyle parenting programmes is INFANT (Infant Feeding Activity and Nutrition Trial), which focuses on parenting skills to support the development of healthy lifestyle behaviours in infancy and employs an anticipatory guidance framework (AGF) [26]. This framework helps the parents manage certain behaviours in advance rather than managing the problems once they have materialised [26]. The modified Play & Grow intervention proposed here will use the same AGF in addressing the eating, sedentary habits of children aged 2 to 4. According to our conceptual model (Fig. 1), the AGF and parenting support theory ‘children’s psychological and behavioural goals, logical and natural consequences and encouragement’ [27], will aid parents in their *general parenting skills* and knowledge on better-practice eating, active play and Connectedness to nature behaviours in children. This will hopefully modify *specific parenting practices* (e.g., eating, active play and Connectedness to nature routines) that will consequently result in improved family lifestyle and child health.

Connectedness to nature/ relatedness is a relatively new concept that, to date, has been investigated primarily in adults [28]. The concept suggests, for example, that engaging in activities in a natural environment can induce a feeling of Connectedness to nature. Exposing preschool children to nature and to thinking about nature in different ways could stimulate Nature Relatedness [29, 30] as a measurable construct. When children are placed in a natural setting, they tend to engage more in active play [15, 17, 18]. As noted above, parents are children’s role models [12], and we therefore believe that connecting parents and other caregivers (particular situation in Hong Kong, which includes grandparents and domestic helpers) with nature will have benefits for their children [12]. Many habits and attitudes are developed early in life, and those related to the natural world are no exception. In fact, children are inherently interested in their environment and in nature in general [21, 31]. We hypothesise that both indoor and outdoor nature-related activities may induce Connectedness to nature and bring about positive changes in both eating and activity habits (short-term outcomes) in preschool children, in turn leading to a healthier lifestyle (long-term outcomes) (Fig. 1).

### The pilot programme Play & Grow

Play & Grow is a 10-week family-based, multi-component healthy lifestyle programme for families with preschool children aged 2 to 4, and was developed and tested in a pilot study (*n* = 39, aged 2.15, SD = .88, retention rate of 93 %) by the PI and her team. The aim of Play & Grow is to encourage healthy eating habits and active play in children from an early age, and to bring about healthy behavioural changes in families. The programme also includes a Connectedness to nature element designed to equip parents with environmental knowledge and skills. The generated pre- and post-test comparison data indicated the effectiveness of the programme (not presented here). A detailed manual and full resource kit were created, and each session consisted of: (i) a 15-min theoretical education component (food, activity, Connectedness to nature), and (ii) a 30-min component on indoor and outdoor nature-related activities, such as playing with objects found in nature and searching for natural treasures (Table 1). Some nature activities were food-related, such as growing plants, creating miniature indoor gardens, and healthy cooking. While the intervention group received the programme as detailed in the resource kit, the control group received an information folder containing government-recommended physical activity and dietary guidelines for children [27]. To enhance participation, all the sessions were scheduled on weekends. The outcome data was collected using a number of scales and questionnaires addessing eating, active play and nature-related habits before and after the programme, and group session debriefings were held at the beginning and end of the programme. The programme proved popular amongst participants and the results demonstrated significant positive changes on a number of health-related outcomes, such as healthier food habits and an increase in caregiver physical activity levels.

**Table 1:** Intervention content and Anticipatory guidance topics, enhanced with connectedness to nature

Session	Activities during the pilot study
1	Welcome and Intro: Setting goals, Communication in the family. Introduce basic concepts regarding parental feeding styles and how these might relate to beliefs about parenting and safety of children. *Connectedness to nature/Outdoor play: active nature games, discovering nature, practicing awareness to sounds, touch, smells, temperature, etc.*
2	Healthy eating: Food groups and reading food labels. How much to eat? Develop parents understanding regarding basic nutrition principles. *Connectedness to nature/Outdoor play: active nature games, discovering nature, practicing awareness to sounds, touch, smells, temperature, etc.*
3	Active play: Methods to encourage active play. Decrease inactive time. Motor skill development for children – the foundation for an active life and safety. Develop of themes/skills regarding: moving for health parents and sedentary behaviours in families. *Connectedness to nature/Outdoor play: active nature games, discovering nature, practicing awareness to sounds, touch, smells, temperature, etc.*
4	Sleeping time: Sleeping friend and sleeping routines. Develop parents understanding regarding sleeping behaviours. *Connectedness to nature/Outdoor play: active nature games, discovering nature, practicing awareness to sounds, touch, smells, temperature, etc.*
5	Fuzzy eating: The outside environment and children. Develop parental skills: how to feed/how to manage food rejection and demands. *Connectedness to nature/Outdoor play: active nature games, discovering nature, practicing awareness to sounds, touch, smells, temperature, etc.*
6	Limit setting: Power struggle. Portion size. Provide parents with understanding about feeding styles and impact on children’s eating. *Connectedness to nature/Outdoor play: active nature games, discovering nature, practicing awareness to sounds, touch, smells, temperature, etc.*
7	Fun with food: Cooking together. To develop understanding about parental modelling of eating, sedentary behaviour and physical activity. *Connectedness to nature/Outdoor play: active nature games, discovering nature, practicing awareness to sounds, touch, smells, temperature, etc.*
8	Encouraging healthy habits: Rules and routines. Throwing, catching, and bouncing skills. Develop skills on providing fail-safe food and activity environments. *Connectedness to nature/Outdoor play: active nature games, discovering nature, practicing awareness to sounds, touch, smells, temperature, etc.*
9	Nature and me: Run & Fun: Promoting PA in Nature. Safety and fun in the nature. Develop parental skills for creating safe outdoor activities in nature environment. *Connectedness to nature/Outdoor play: active nature games, discovering nature, practicing awareness to sounds, touch, smells, temperature, etc.*
10	Farewell and graduation: Summary. *Connectedness to nature/Outdoor play: active nature games, discovering nature, practicing awareness to sounds, touch, smells, temperature, etc.*

### Objectives

The main objective was to test the hypothesis that the proposed healthy lifestyle intervention program Play & Grow, will improve stated outcomes. The effectiveness of Play & Grow intervention, will be tested by conducting a randomised controlled trial (RCT), enhanced with the novel connectedness to nature element, and evaluating immediate and long-term health effects on participating children's active play/sedentary behaviours, zBMI eating habits (Stage 2).

- To validate the newly created NRS on the preschool population of Hong Kong (Stage 1)
- To improve general parenting practices (encouragement, positive involvement, problem solving, knowledge of child nutrition and active play, age-appropriate activities and attitudes to nature); and specific parenting practices such as outdoor playtime routines, meal and snack routines, environmental practices and (Connectedness to nature) (Stage 2)
- To test whether Connectedness to nature encourages healthy lifestyle routines and positively influences healthy eating and active playtime in children aged 2 to 4 in Hong Kong (Stage 2).
- To test if the key elements of the Play & Grow programme; that is, offering anticipatory guidance [26] and emphasizing parenting skills [23], will be beneficial for the outcomes of the intervention. Further, we assume that the added novel element, Connectedness to nature, will boost the programme and have additional beneficial effects on the children's well-being as a result of increased outdoor time and participation in activities related to nature, active play and food.

## Methods and design

**Fig. 2:**
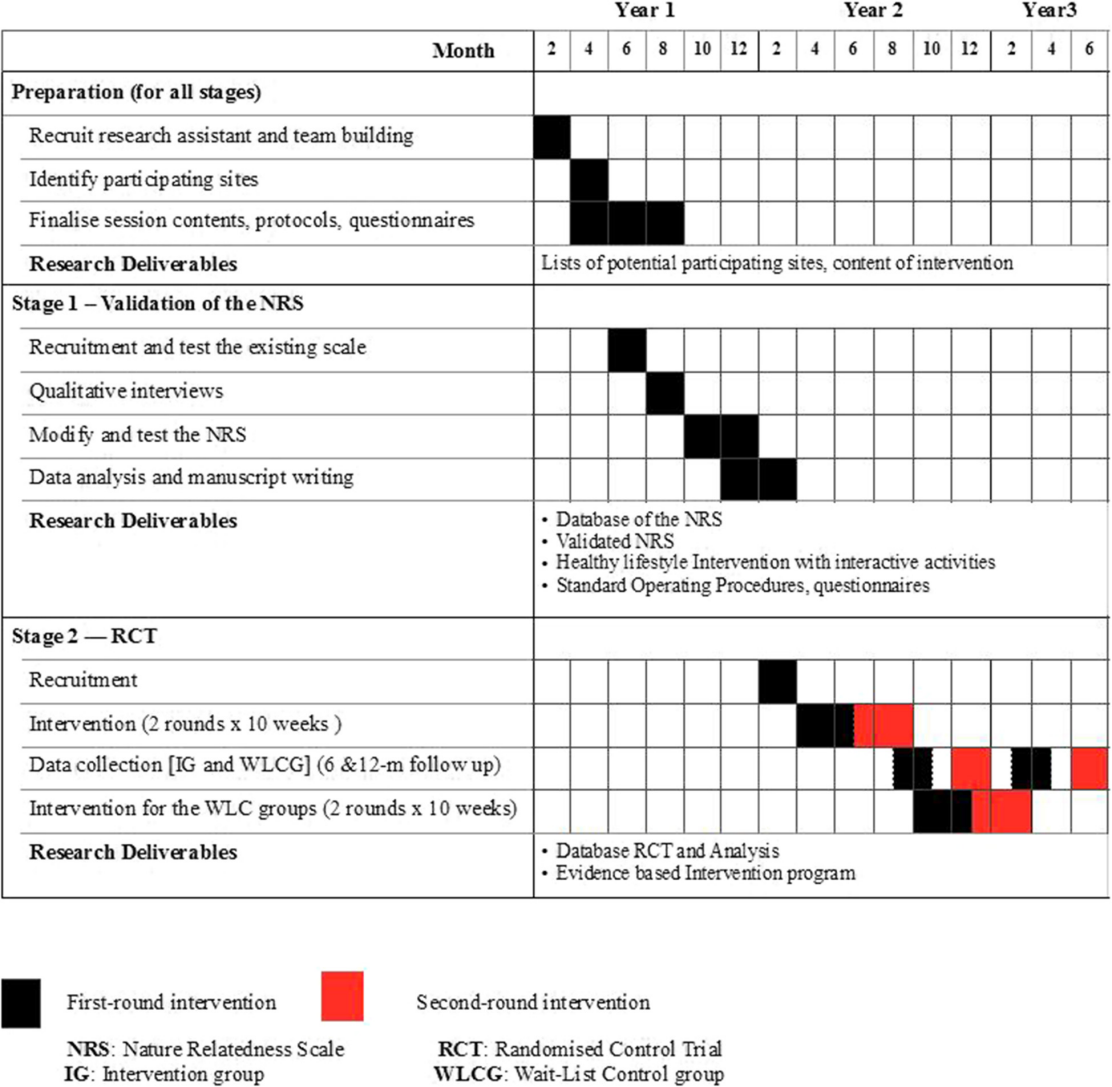
Tentative project timeline

The objectives of the proposed project will be achieved in two stages over a 30-month period (Fig. 2) in Hong Kong. Firstly, to create a valid measurement tool for the novel Connectedness to nature intervention element, a Nature Relatedness Scale (NRS) will be validated and tested for reliability (Stage 1). To measure the effectiveness of the early-intervention Play & Grow programme, a RCT will be conducted (Stage 2). In order to investigate which particular components of the Play & Grow were most/least effective, the data from the RCT will be used to perform a mediator study at a later stage.

### STAGE 1. NRS for preschool children – Constructing and evaluating a new measuring instrument

#### Hypothesis

The Nature Relatedness Scale (NRS) for preschool children is a short, reliable, and valid scale for measuring the environmental behaviours of preschool children aged 2–4 years in Hong Kong.

An understanding of children’s nature relatedness becomes more critical as the casual interaction with nature in daily life becomes less obvious. As none of the existing methods for measuring children’s attitudes and behaviours towards environment and nature have been assessed in preschool children, little is known about how they perceive and interact with the natural environment. This baseline is crucial to understanding whether health-promoting interventions targeting Connectedness to nature can be effective. We modified an existing scale for older children in our recent Play & Grow pilot study [32]. Overall, the reliability and construct validity indicated very low applicability to Hong Kong pre-schoolers (Cronbach’s alpha and inter-item correlation of most dimensions, e.g. < 0.50). In the proposed study, we will therefore employ a mixed-method approach, including the above mentioned Play & Grow pilot test (*n* = 39), qualitative interviews with parents (*n* = 10), further modification of the items, and a test of the final short 20-item NRS on children of 2 to 4 years of age (*n* = 100) in 2 different socioeconomic settings in Hong Kong. The qualitative data will lead to construction of an item pool with items representing four theoretical constructs of Nature Relatedness: (1) enjoyment of nature, (2) empathy for nature, (3) sense of responsibility, and (4) awareness of nature. We will collect a sub-sample from 110 children in this age group and explore the factor structure. Participants will be recruited through the Active Health Clinic, HKU. In addition to the Nature Relatedness items, we will include attitude to nature, enjoyment/happiness, psychological health outcomes, and subjective well-being. The Pediatric Quality of Life Inventory 兒科生活品質問卷 (Varni JW, Version 4.0- translated) [33] will be used in the survey to test the criterion validity of NRS for preschool children.

#### Analysis

We will employ exploratory factor analysis to examine the factor structure of the NRS for preschool children. This analysis will reveal the number of factors naturally present in the data, and allow us to examine whether the responses of the items are loaded consistently on the corresponding factors. The appropriateness of factor analysis will be tested and the convergent validity of the scale will be examined by Cronbach’s alpha. The criterion validity of the scale will be examined by correlating the constructs of the scale to attitude to nature, enjoyment/happiness, psychological health, and subjective well-being; that is, outcome variables theoretically positively related to Nature Relatedness [32].

### STAGE 2. Play & Grow, RCT

**Fig. 3:**
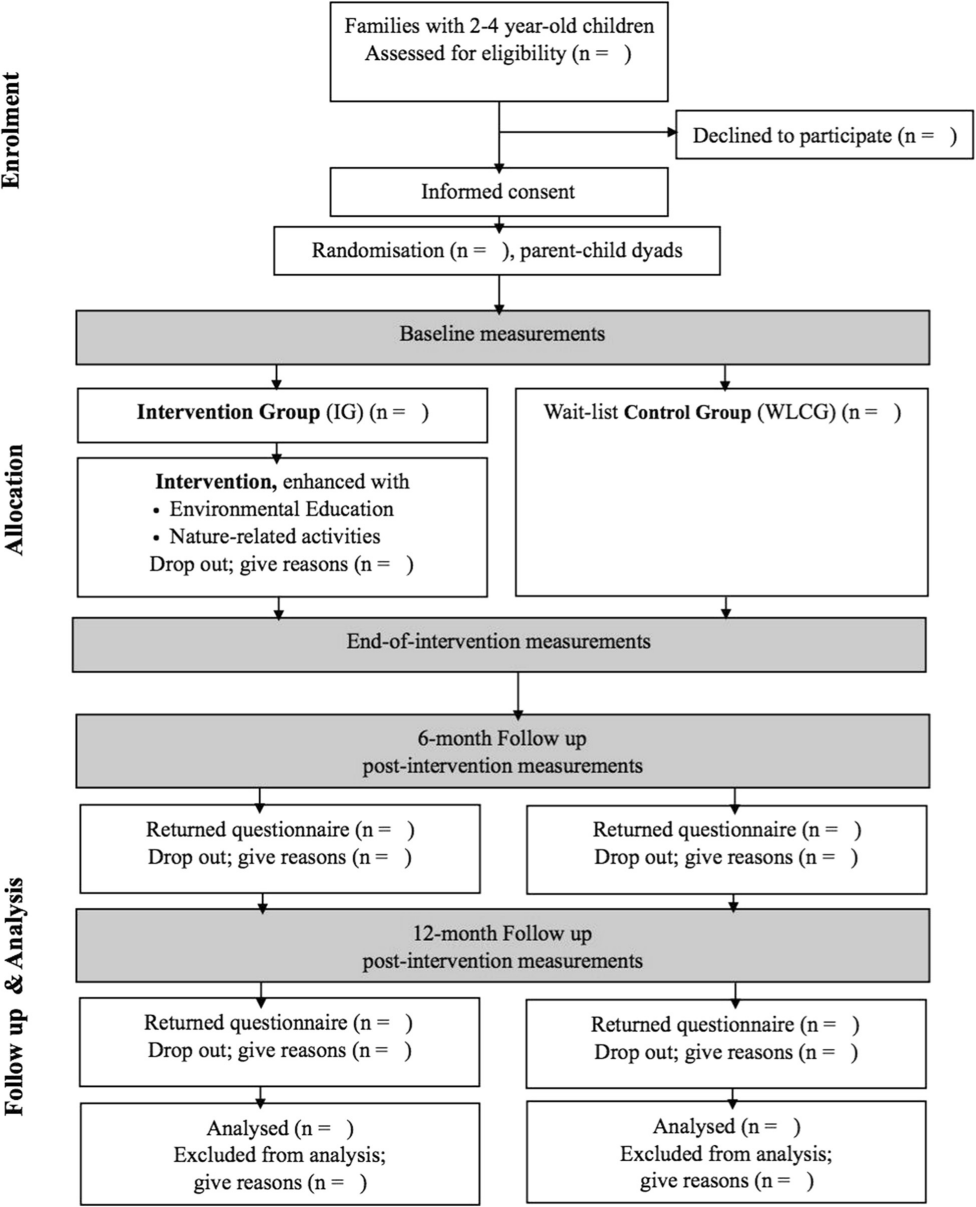
RCT, Study design

In the RCT, the families with 2 to 4 year old children will be randomly allocated to either the intervention group (IG) or waiting-list control group (WLCG). The intervention will comply with CONSORT [34] (Fig. 3). The program is based on the recently completed Play & Grow pilot study, which utilised elements of the three successful healthy lifestyle interventions EarlySTOPP [22], INFANT study [23] and MEND [35], with the addition of the novel Connectedness to nature element.

#### Hypotheses

After completing the Play & Grow program (10-week, 6 and 12 months), *children* in the IG, when compared with the WLCG, will: (1) eat healthier, have a higher responsiveness to satiety, be less fussy with food, have less food neophobia; eat more fruit, vegetables and less obesogenic foods; (2) be more active, less sedentary and (3) have a higher Connectedness to nature, reflected in increased time spent outdoors. The *parents* in the IG will: (1) know more about healthy eating, active play and Connectedness to nature; and 2) gain *general* and *specific* parenting practices, including role-modelling for healthy habits.

#### Participants and randomization

Two to four year old children and their English-speaking parents will be recruited through kindergarten advertisements and via the HKU website. For continuity of delivered message, both parents in two-parent families will be invited to join. According to our pilot experience, however, we expect mostly mothers and domestic helpers to attend. The fathers will be welcome as well, but the same caregiver should fill the questionnaires. We set up a requirement of 80 % participation attendance of the same caregiver. Given the Play & Grow pilot data, 100 children in each group (*p* < 0.05 and power 0.8) will be needed to detect changes between the intervention and control groups. The WLCG children will be offered the Play & Grow programme at study completion (Fig. 3).

#### Power and sample size

The primary outcomes for this intervention relate to eating, active play and Connectedness to nature habits. The sample size calculation is based on a similar study conducted in New Zealand, MEND 2–4, which provides relevant parameter estimates for children aged 2 to 4 years of age [35, 36]. As there have been no similar interventions in this age group in Hong Kong, we adpoted the MEND design suggestion and calculated the sample size first of all, in regards to our primary outcomes (for example, 25 % increase in vegetable consumption), hence 100 families in each group will be required for a power of 0.8, α = 0.05 [35]. Based on the pilot data, and the dropout rate of 20 %, we intend to recruit 240 families. This sample size will be sufficient to detect medium effect sizes (power of 0.8 at α = 0.05) in our the secondary outcomes [37]. To increase retention in the study, we will build connections with the participants through a blog created for the Play & Grow pilot, and send birthday cards to each child participant.

#### Intervention

Each session will present, discuss and enact a certain parenting practice (Table 1). The Play & Grow will have educational strategies including instructions, parental peer support and group discussions, and homework tasks, in accordance with the elements developed in our Play & Grow pilot study. Each session will comprise: (i) 15 min of guided active play involving both children and parents; (ii) 15 min of interactive education and skill development for parents; simultaneous supervised active play with foods for children, to promote acceptance of vegetables, and (iii) 15 min of guided active nature games outdoors, involving both children and parents. The sessions will incorporate a lifestyle component, for example: eating, active play and connectedness to nature). These will target the parents’ knowledge and skills on how to introduce and maintain their child’s correct lifestyle routines. A group leader and co-leader with healthcare backgrounds (and trained by the PI during the Play & Grow pilot study) will facilitate the sessions involving 4 to 5 parent-child dyads.

#### The novel Connectedness to nature element

The most appropriate way of instilling habits related to nature is to stimulate children's natural curiosity as early in life as possible. This could be done by involving caregivers and educating them about nature-related activities [30, 38–41]. In adults, research demonstrates a correlation between learning about the nature and the length of time spent in nature [42]. Connectedness to nature is described as including basic environmental knowledge, environmental sensitivity and awareness, as well as a commitment to protect nature [28]. In the proposed intervention, we will employ environmental education and nature-related activities to help participating families develop skills conducive to improving playtime and eating habits in children. Examples of the additional elements that make up an 1/3 of the programme are: i) inclusion of active nature games outdoors that involve both children and parents focusing on motor skills, discovering nature, practicing awareness to sounds, touch, smells, temperature, ii) group activities with other families and members, practicing nature related activities, iii) nature homework with follow-ups (collect objects found in nature and make art or grow their own plants), iv) environmental care training (paper waste, water saving, recycling), and v) studying new vegetables and cooking together with them (avocado, using the avocado to make a boat, turning the avocado into guacamole, etc.).

### Primary outcomes and their assessments

Measurements will be conducted prior to and after the 10-week intervention, with further follow up assessments at 6 and 12 months in order to observe both the short and long term outcomes (Table 2). Although many variables will be measured in both children and participating parents, we carefully chose a few questionnaires, which covered multiple variables.

**Table 2:** Intervention outcome instruments

Intervention Outcomes	Source	Baseline	End-of-intervention	6-month Follow-up	12-month Follow-up
Parent					
General Parenting Practices					
Knowledge of children’s:					
Feeding	PCFQ	✕	✕		
Nutrition	NKQ	✕	✕		
Active play	Pre-PAQ	✕	✕		
Connectedness to nature	NRS (self-developed)	✕	✕		
Correct practices:					
Feeding	CFPQ	✕	✕	✕	✕
Nutrition	NKQ	✕	✕	✕	✕
Active play	Pre-PAQ				
Connectedness to nature	NRS (self-developed)	✕	✕	✕	✕
Parental self-efficacy for correct practices	INFANT scale	✕	✕	✕	✕
Parental modelling of healthy habits, diet, active play, connectedness to nature	INFANT scale	✕	✕	✕	✕
Limit setting	Self-developed scale	✕	✕	✕	✕
Specific Parenting Practices					
Diet	CEBQ	✕	✕	✕	✕
Active Play	IPAQ, Pre-PAQ	✕	✕	✕	✕
Sedentary time	Pre-PAQ	✕	✕	✕	✕
Connectedness to nature	NRS (self-developed)	✕	✕	✕	✕
Child					
Eating Habits	EPAQ	✕	✕	✕	✕
Physical Activity	Pre-PAQ	✕	✕	✕	✕
Sedentary Behaviour	Pre-PAQ	✕	✕	✕	✕
Food Neophobia	PCNS	✕	✕	✕	✕
Connectedness to nature	NRS (self-developed)	✕	✕	✕	✕

Please see detailed references in proposal’s text

#### Eating habits

A short validated Eating and Physical Activity Questionnaire (EPAQ) will be used [43] and The Children’s Eating Behaviour Questionnaire (CEBQ), previously validated by the PI in China [44], will be used to assess the children's eating styles/habits.

#### Physical activity and sedentary time

Physical Activity Questionnaire for Preschool-aged Children (Pre-PAQ®) [45] valid for young children’s activity habits [46].

#### Nature relatedness

Connectedness to nature in children will be measured using a short, age-adjusted NRS scale that will be validated in Stage 1. The scale uses a number of specific questions about environmental sensitivity, distance from home to natural areas, how often and how long the family spends in nature and what nature-related activities are done, including questions on recycling, garbage sorting and saving water.

### Secondary outcomes and assessments

Many of the MENT study assessments will be used in this RCT, since they are validated for this age and outcomes [46]. These include *Parental knowledge of nutrition* (Nutrition Knowledge Questionnaire (NKQ) [47]), *Parental behaviours related to feeding, eating and physical activity* (The Preschool Child Feeding Questionnaire (PCFQ) [48] and the Child Feeding Questionnaire (CFQ) [8]); Validated Active Australia Survey [49]; Parental encouragement and parent cognitions: 5-point Likert scales, Pliner’s Child Neophobia Scale (PCNS) [50].

#### Anthropometry

Anthropometric data will be collected and aggregated according to the standard techniques and age-adjusted references [36, 51]. The Body Mass Index (BMI, kg/m2) will be calculated for each participant, BMI-for-age z-scores will be used to for children, following the WHO recommendations for children by age and gender [52, 53]. Parental age, socioeconomic status and education level will be assessed according to the study protocols developed for Hong Kong and tested during the Play & Grow pilot project.

#### Analysis

To describe the demographic and other subject characteristics and to evaluate the distributions the descriptive statistics will be applied. We will use an intention-to-treat statistical approach. Regression models will address the effect of the intervention on the studied outcomes (Multivariate Analysis of Variance, multiple regressions, controlling for baseline and other covariates). Linear mixed modelling will be used to analyse physical activity/sedentary behaviour changes, dietary habits, zBMI and food neophobia. Our sample should be adequate for running a two-way (2x3) repeated measures ANOVA for our study, but if the sample is not normally distributed, a non-parametric two-way ANOVA, namely the adjusted rank transform ANOVA, to analyse the between-subject effect, within-subject effect, and interactions effects will be used [54].

## Discussion and potential implications

Firstly, this study will develop, test and validate in Hong Kong an instrument to measure Connectedness to nature in preschool children in Hong Kong. This scale could be the first promising tool for understanding and predicting health-related environmental attitudes and behaviours, and how these change over time. It has the potential to be used for establishing causal associations between an intervention and an outcome and evaluation of health-promoting interventions in general.

To the best of our knowledge, this type of intervention is novel in Hong Kong and internationally. The results are expected to contribute to the knowledge on physical activity and diet in preschool children and by introducing a new environmental element, Connectedness to nature, to the healthy lifestyle recommendations.

## Abbreviations

AGF, anticipatory guidance framework; BMI, body mass index; CFQ, child feeding questionnaire; IG, intervention group; INFANT, infant feeding activity and nutrition trial; MANOVA, multivariate analysis of variance; NKQ, nutrition knowledge questionnaire; NRS, nature relatedness scale; PCFQ, the preschool child feeding questionnaire; PCNS, Pliner’s child neophobia scale; RCT, randomised controlled trial; WLCG, waiting-list control group.
